# “That’s enough patients for everyone!”: Local stakeholders’ views on attracting patients into Barbados and Guatemala’s emerging medical tourism sectors

**DOI:** 10.1186/s12992-016-0203-7

**Published:** 2016-10-07

**Authors:** Jeremy Snyder, Valorie A. Crooks, Rory Johnston, Alejandro Cerón, Ronald Labonte

**Affiliations:** 1 Faculty of Health Sciences, Simon Fraser University, 8888 University Dr, Burnaby, BC V5A 1S6 Canada; 2 Department of Geography, Simon Fraser University, 8888 University Dr, Burnaby, BC V5A 1S6 Canada; 3 Department of Anthropology, University of Denver, 2199 S. University Blvd, Denver, CO 80208 USA; 4 Faculty of Medicine, University of Ottawa, 75 Laurier Ave E, Ottawa, ON K1N 6N5 Canada

**Keywords:** Medical tourism, Latin American and Caribbean (LAC) region, Guatemala, Barbados, International patients, Comparative case study

## Abstract

**Background:**

Medical tourism has attracted considerable interest within the Latin American and Caribbean (LAC) region. Governments in the region tout the economic potential of treating foreign patients while several new private hospitals primarily target international patients. This analysis explores the perspectives of a range of medical tourism sector stakeholders in two LAC countries, Guatemala and Barbados, which are beginning to develop their medical tourism sectors. These perspectives provide insights into how beliefs about international patients are shaping the expanding regional interest in medical tourism.

**Methods:**

Structured around the comparative case study methodology, semi-structured interviews were conducted with 50 medical tourism stakeholders in each of Guatemala and Barbados (*n* = 100). To capture a comprehensive range of perspectives, stakeholders were recruited to represent civil society (*n* = 5/country), health human resources (*n* = 15/country), public health care and tourism sectors (*n* = 15/country), and private health care and tourism sectors (*n* = 15/country). Interviews were transcribed verbatim, coded using a collaborative process of scheme development, and analyzed thematically following an iterative process of data review.

**Results:**

Many Guatemalan stakeholders identified the Guatemalan-American diaspora as a significant source of existing international patients. Similarly, Barbadian participants identified their large recreational tourism sector as creating a ready source of foreign patients with existing ties to the country. While both Barbadian and Guatemalan medical tourism proponents share a common understanding that intra-regional patients are an existing supply of international patients that should be further developed, the dominant perception driving interest in medical tourism is the proximity of the American health care market. In the short term, this supplies a vision of a large number of Americans lacking adequate health insurance willing to travel for care, while in the long term, the Affordable Care Act is seen to be an enormous potential driver of future medical tourism as it is believed that private insurers will seek to control costs by outsourcing care to providers abroad.

**Conclusions:**

Each country has some comparative advantage in medical tourism. Assumptions about a large North American patient base, however, are not supported by reliable evidence. Pursuing this market could incur costs borne by patients in their public health systems.

## Introduction

Many governments in the Latin American and Caribbean (LAC) region are actively promoting their health services to private international patients. More commonly called ‘medical tourists’, these are patients who travel internationally with the intention of receiving medical care paid for out of pocket. Patients have long crossed national borders to go to nearby countries within the region in order to access medical care, particularly as several LAC nations in Central America and the Caribbean consist of small states that are unable to provide a full range of medical services domestically [1, 2]. More recently, emphasis has been placed on promoting medical travel from across the world to the LAC region, with particular emphasis on attracting ‘customers’ from the United States (US) and Canada [3, 4].

Well established medical tourism destinations in the LAC region include Cuba, Costa Rica, and Panama [5]. Hospitals in these established medical tourism destinations have long provided care to patients from throughout the LAC region, having only relatively recently expanded their marketing to international patients beyond the region [1]. Recent years have seen a number of other LAC countries express interest in developing medical tourism, most of which are at an early stage of sector development. For example, in 2014 a 104 bed hospital focused on performing heart and cardiac surgeries for international patients opened in the Cayman Islands [6]. The Bahamas hosted American investors in 2013 who were interested in developing a controversial US$200 million hospital that would offer a range of surgical procedures for patients from North America [7]. Belizean physicians and tourism operators recently formed the Belize Medical Tourism Association and are actively seeking investment in this sector from the US [8]. In Jamaica, there are plans to open a US$170 million 50–75 bed facility focusing on cosmetic, bariatric, and dental services, expanding in future years to a 200 bed multi-specialty hospital [9]. Colombia is seeking to increase the number of facilities catering primarily to international patients (currently there are over twenty providing a range of tertiary services to around 30,000 medical tourists annually), with government incentives that include generous tax holidays, enhanced English language training for health workers and efforts to increase the number of medical specialists [10]. The Turks and Caicos hosts some Canadian orthopedic surgeons who in recent years have started to bring their patients there for treatment in order to avoid wait times for care in Canada [11]. These are but a few specific examples of types of medical tourism initiatives emerging in the LAC region. Governments of these countries and private sector partners must work to establish their ‘brand’ as a viable medical tourism destination, which requires consideration of who their desired international patient base is and how they will compete for attracting these patients, including compared to others in the LAC region.

On the one hand, medical tourism has the potential to diversify the economies of LAC destination countries through recruiting privately-paying international patients by providing more specialist services than the domestic market can support alone. These expanded health services are thought to help retain both local patients who currently travel internationally for care as well as the high numbers of skilled health workers who emigrate for more professionally rewarding or lucrative posts elsewhere [12]. However, it is unclear if these outcomes will be realized for the many LAC countries competing with each other to develop medical tourism sectors, particularly when success is predicated on luring what is perceived to be a large and lucrative North American market to their shores [13]. On the other hand, if the patient flows hoped for by these countries do not materialize, there is a danger that considerable time and resources will have been wasted that could otherwise have been used within the domestic health system or to develop other economic opportunities. Given the many LAC governments and hospitals vying for the same North American market, it is likely that some, at least, of these countries will not be successful in their pursuits.

Here we examine the issue of how local stakeholders in two LAC countries, Barbados and Guatemala, understand who their target medical tourists are by presenting a thematic analysis of one hundred purposively sampled key informant interviews conducted in these sites. As we show in this paper, the governments and some private health care providers in both countries are beginning to compete with other countries in the LAC region to attract international patients. In this novel analysis we examine the issue of who their target patients are and, more importantly, local stakeholders’ rationales for seeking (or not seeking) particular patient groups. Several recently published reviews have established that significant knowledge gaps that remain about medical tourism [14, 15]. We believe that this analysis addresses several such gaps. First, we provide some of the first empirical insights about the development of the medical tourism sector in Guatemala while complementing the few studies previously conducted in Barbados [1, 3, 16–19], all of which contribute new first-hand insights to the global dialogues about the health equity impacts of the medical tourism industry (e.g., [20, 21]). Second, we provide on-the-ground insights from some local stakeholder groups that have rarely been consulted about their perspectives on medical tourism sector development, such as community organizations and other civil society representatives. Third, and most importantly, we show that destination country stakeholders do not view medical tourists as a homogenous group. Although this might sound logical, much of the scholarly literature on medical tourism glosses over such differences, and never has an analysis explicitly interrogated them nor situated them within the context of local and regional medical tourism sector growth. We show throughout this paper that some medical tourists are thought to be more desirable than others by destination countries, or are thought to be more likely to travel to particular countries than others, and the basis for this reasoning.

## Background

Guatemala, located on the Central American isthmus and bordering the Caribbean Sea, is a lower-middle income LAC region country with a land area of 108,889 km^2^ and a population of 15.5 million people [22]. Guatemala has a small number of highly resourced private hospitals and what is widely thought to be an under resourced public health care system. There is a great range in the quality of care available throughout the country and most Guatemalans rely on charitable or public hospitals for medical care [23]. Barbados is a small island state in the Eastern Caribbean. A high-income country, it is far smaller than Guatemala with only 431 km^2^ of land and a population of 285,000 people [22]. Barbados’ health system provides universal coverage for public medical services to citizens, while private clinics and a small hospital provide faster access to patients with private insurance or the ability to pay directly [24]. While the health systems of Barbados and Guatemala contrast with one another in their organization and capacity, both are at the early stages of orienting themselves towards exporting their private health care services to the international market. In the remainder of this section, we provide a brief overview of the private and public sector activities occurring in each country that are acting to promote medical tourism.

In order to promote its medical tourism sector, in 2011 Guatemala’s Tourism Commission for Health and Wellness started undertaking trade missions abroad and attending national and international trade fairs aimed at the medical tourism market. These efforts were coupled with familiarization tours of the Guatemalan private medical sector for foreign medical tourism brokers, insurance agents, and the press. The target market for these early initiatives were expatriate Guatemalans and other Latin Americans living in the US, as well as baby boomers and self-insured businesses in the US without existing connections to the LAC region [25]. The Guatemalan Association of Exporters (AGEXPORT) separately conducted a study of the Guatemalan private health care sector compared to LAC regional competitors in 2013. This study determined that dentistry, human reproduction, and ophthalmology were Guatemala’s most competitive and viable sectors for medical tourism [26].

The focus of Guatemala’s medical tourism sector development is on making better use of existing capacity rather than building new facilities specifically targeting high-paying international patients. Medical tourism facilitation companies aiming to bring American patients to Guatemala tout the cost savings when compared to the US and the high quality of care available at some private hospitals [27–29]. In order to make their facilities more appealing to international patients, some facilities in Guatemala are seeking Joint Commission International accreditation and bi-lingual nursing training opportunities [23]. Medical tourism brokers advertise Guatemala as a medical tourism destination online, emphasizing its cost-competitiveness and quality to potential clients in North America. AGEXPORT has actively promoted medical tourism from the US by attempting to partner with US employers and banks to facilitate insurance coverage and payments for medical treatments [23]. However, the distance of Guatemala from the US when compared to other LAC regional competitors has been highlighted as a disadvantage for being able to develop a robust medical tourism sector [30].

As with Guatemala, Barbados is actively seeking private patients from outside the Caribbean. In 2008, the government of Barbados created a health and wellness taskforce that intended to develop the medical tourism sector with an aim toward attracting patients from outside of the region. Barbados also hosted conferences promoting international health and wellness tourism in 2008 and 2010 [24]. A Health and Wellness Taskforce, formed in 2008 by the Ministry of Health, was tasked during this period with incentivizing foreign investment in Barbados’ medical tourism sector. These incentives include tax exemption for medical equipment imports, exemption from real estate taxes, and suspension of costs associated with starting and reorganizing new businesses. This task force also sought to promote Barbados’ brand as a safe, high quality destination for medical treatment in North American and the EU. This was attempted through promotional activities online, trade missions, referral networks with health care providers, and promotion of positive news stories on Barbados’ health sector in international media [24].

To date, medical tourism activities largely focus on the Barbados Fertility Centre, a small reproductive health clinic that draws on patients from the Caribbean as well as the US, Canada, and United Kingdom (UK) [3, 31]. This clinic reports a 33 % growth in patients since 2009 despite the economic downturn and drop in traditional tourism during this time [32]. Barbados has also hosted several failed attempts at medical tourism, including a stem cell clinic and several proposed international hospitals that have not materialized [24]. A proposed new facility, American World Clinics (AWC), is seeking to target the North American market on a much larger scale than that seen with the Barbados Fertility Centre by building a 50 bed multi-specialty hospital on the site of a former private hospital [33]. This proposed facility, financed by American investors, is the result of an active push into medical tourism by the Barbadian government which sought bids from international investors to develop the former hospital site to primarily serve medical tourists [24]. The future of this project is uncertain, however; originally scheduled to open in 2013, the project has been delayed [5].

As outlined above, the medical tourism industry in the LAC region is at present rapidly developing and volatile. However, it is not clear if the push to develop this sector, with its focus on accessing privately paying patients from the North American market, is the result of a realistic assessment of the potential of these markets or the outcome of industry hype and a fear of being left out of the competition for foreign investment in health services. In the remainder of this paper we examine what target patient markets are driving local stakeholder perceptions of medical tourism’s potential in the LAC region, with a focus on Barbados and Guatemala, and how these perceived markets are shaping planning and development of the sector.

## Methods

The goal of this qualitative study, guided by comparative case study methodology [34, 35], is to examine the health equity impacts of medical tourism in particular destination countries in the LAC region, with a focus on identifying negative and positive health system and policy changes related to public health care, private health care, health human resources, investment, and domestic government involvement. This goal emerged in direct response to the health equity debates and literature that have extensively outlined the knowledge gaps that exist regarding the local impacts of medical tourism in destination countries and the lack of empirical research available to support the various positions taken by stakeholders in these debates [20, 36–38]. The analysis presented in this article contributes to achieving our overall study goal by exploring the ways in which emerging medical tourism destinations – using the cases of Barbados and Guatemala, both of which have nascent medical tourism sectors with strategic plans for enhancement - envision this sector unfolding and who the likely international patient inflows will be, wherein the composition of these inflows directly impacts the types of local transformations that are needed in order to attract these patient markets.

### Recruitment

Following receipt of ethics approval, we sought to purposefully recruit 50 key informant interviewees in each of Barbados and Guatemala. In order to capture both breadth and depth of perspective we sought to identify key informants who represented specific sectors, and looked to speak with five civil society representatives (e.g., non-governmental organizations, local chapters of international organizations, community groups, media), 15 health human resources representatives (e.g., health workers, medical education professionals, health worker union representatives), 15 government or public health care/tourism sector representatives (e.g., employees of government ministries, hospital and health system administrators, tourism officials, investment sector representatives), and 15 private health care/tourism representatives (e.g., tourism consultants, owners/administrators of private health care clinics, private investment experts, investors) in each country. Note that in this article we do not provide a more detailed breakdown of participants by job type or sector because of the high risk for identification. Potential participants were identified simultaneously using multiple channels: (1) searching media coverage for names of key individuals; (2) reviewing industry reports; (3) speaking with members of our professional networks in each country; (4) identifying specific offices/organizations of interest; and (5) asking participants to share information about the study with others in their networks who might be interested in participating.

After potential participants were identified an e-mail containing information about the details of the interview and study was sent along with a request to participate. All potential participants were invited to take part in the study because of their professional and practical knowledge, and in some cases this meant speaking to people who were aware of issues highly relevant to medical tourism but had no detailed knowledge about medical tourism. Because of this, these initial e-mails were tailored to each individual, explaining why his/her perspective was particularly useful to the study. In some cases this initial contact was made by phone or in person. Those interested in participating in the study were asked to reply by e-mail or phone to express this interest, after which an interview was scheduled at a time and location of the participant’s preference. In instances where interviews were scheduled far in advanced, reminder messages were sent to confirm the time and location. In some cases interviews were scheduled by phone in order to accommodate availability or travel schedules.

### Data collection

Interviews in Barbados were conducted in English throughout the country between May, 2013 and February, 2014 by a team of two researchers and two highly trained research assistants. Interviews in Guatemala were conducted in Spanish in Guatemala City and Antigua between June and December, 2013 by a team of two highly trained research assistants. Interviews typically lasted between 45 and 90 min after informed consent was achieved and were most commonly conducted one-on-one, though in some instances group interviews were scheduled based on participants’ preferences.

Interview questions were developed following an extensive review of the literature about the health equity impacts of medical tourism [36], the development of highly detailed background reports about the development of the medical tourism sectors in Barbados and Guatemala [23, 24], and a review of insights gleaned from our earlier pilot research in Barbados [16, 18, 19]. Interviews in both countries were conducted using a single guide that was organized around a set of common questions asked of all interviewees and groupings of tailored questions that were asked according to participants’ expertise. The common questions probed overall knowledge of medical tourism and local health and health system challenges and opportunities as well as participants’ professional backgrounds. Tailored questions explored the domains of health human resources (e.g., What changes, if any, have been implemented in health worker education programs specifically in response to the development of medical tourism here?); domestic government involvement (e.g., What responsibilities does the government hold towards the development of the medical tourism sector here and to medical tourism facilities?); public health care (e.g., Are you aware of any ways in which existing public health care facilities are interested in expanding their services to treat medical tourists?); private health care (e.g., Are private clinics or hospitals organizing, advocating, or lobbying to alter public policy to support or promote medical tourism here?); and foreign investment (e.g., What barriers to private investment in health services exist here? And are there any that are particularly imposing to foreign investors?).

### Data analysis

Interviews were recorded digitally. Those conducted in English were transcribed verbatim. Those conducted in Spanish were simultaneously transcribed and translated by a group with possessing the necessary language skills. Upon completion of data collection, a group of the lead investigators independently reviewed selected transcripts in order to identify emergent themes in preparation for thematic analysis, a technique that involves categorizing data into themes (units identified from patterns in the dataset) and contrasting these themes against both the study objectives and existing literature in order to glean new insights [39]. Following this, a meeting was held to discuss coding scheme development, emergent themes, and analyses worthy of being pursued. After this meeting one investigator drafted a proposed coding scheme that inductively and deductively captured the themes and analyses identified in the meeting. Feedback on the scheme was then sought from the lead investigator in order to ensure it adequately captured what the team had agreed to examine. Next, the 100 transcripts were uploaded into NVivo in preparation for coding, after which the investigator who devised the scheme and a research assistant familiar with the study each coded the same 5 transcripts in order to assess the integrity of the scheme. Following this, and with the input of the lead investigator, the coding scheme was revised in order to ensure more consistent interpretation of each code and reduce redundancy. The full dataset was then coded by a single investigator in order to enhance consistency and overall rigour.

The themes reported on in the current analysis were identified through a collaborative, iterative process. After the 100 interviews were coded, the coded data central to this analysis were extracted and reviewed in full by the lead author. Extracts that best characterized the agreed upon themes were compiled and shared among the team so as to ensure consensus on the scope and scale of their interpretation and to contrast emerging issues against what is known in the existing literature. Throughout the findings section we incorporate direct quotes selected from these same extracts in order to allow the participants’ voices to speak.

## Results

Participants in both Barbados and Guatemala identified two broad groups of patients that are being targeted in developing their medical tourism sectors. The first of these are international patient groups who are readily accessible because of their established inflows into the countries, including members of the Guatemalan diaspora, repeat visitors to Barbados, and LAC regional patients. Each country is also planning to seek out new traveler inflows as international patients, envisioned as being drawn largely from continental North American and, to a lesser extent, European patients with no existing ties to the region. In this section we examine participants’ consideration of each broad inflow, articulating their rationales for seeking particular groups within these inflows and their thoughts regarding the viability of treating such patients as their local medical tourism sectors develop.

### Targeting established inflows as future medical tourists: Guatemala’s diaspora

Guatemalan participants flagged the Guatemalan diaspora as an important existing source of international patients for private health providers, this being an inflow that many thought should be targeted for expansion as the country’s medical tourism sector grows. One participant described this group as coming “*from up north to visit their family. Once they are here they take advantage of the opportunity and seek medical services*.” Although one participant indicated cases of these individuals traveling three times a year for regular medical and dental check-ups, it was more commonly suggested that such patients could be encouraged to seek such routine medical care once a year. Existing Guatemalan diaspora patients were cited as coming from neighbouring countries in the LAC region as well as the US, Canada, and Italy. Some participants expressed reservations about the desirability of diaspora patients in the medical tourism sector as they are seen to have a less positive economic impact on Guatemala than other patients due to their roots in the country: “*What happens is that if you bring a Guatemalan that lives over there [abroad], they will come and stay at a family member’s house, and will eat there. If you bring an American or anyone from anywhere, they will stay at the hotel, use the taxi, he will go to buy at Antigua* [a holiday town], *will eat at restaurants*.” For this reason, it was felt, medical tourism operators should not be satisfied solely with the diasporic market, but should nonetheless seek to increase this existing patient inflow.

Members of the Guatemalan diaspora were thought to be motivated to access care in the country while abroad due to cost savings as “*the United States is too expensive.*” Wait times for care were also cited as a motivation for traveling to Guatemala as “*back in Italy, [you] make an appointment and [you] get it six months later*.” Aside from the costs and wait times that would encourage such individuals to continue to access private medical care in Guatemala, the Guatemalan diaspora was also seen as a desirable group of current and future medical tourists “*because [the patient] knows that doctors are good and he will have a good treatment*.” Whereas many participants raised concerns that actual and perceived violence in Guatemala would dissuade potential medical tourists not familiar with the country, “*if your base population … is made up by Guatemalans who are residents of the US, you don’t have to convince them of coming here, because of the violence in the country and etc. They already travel to Guatemala!*” It was pointed out that successfully recruiting and treating members of the Guatemalan diaspora for medical care could ultimately, and quite strategically, lead to the development of new patient inflows as they could spread the word about the high quality of care in Guatemala, thereby encouraging others not of Guatemalan descent.

### Targeting established inflows as future medical tourists: Barbados’ recreational tourists

While the medical tourism stakeholders we spoke with in Barbados did not raise emigrant Barbadians as a potential market for the country’s nascent medical tourism sector, they did see regular visitors to Barbados as an easily accessible target market. Barbados’ status as a destination for cruise ships was noted as creating the potential for a larger patient market. If the workers and possibly passengers from these ships could be enticed to access Barbados’ private medical facilities, “*it could be a key thing for us.”* Another group of regular visitors to the country that participants thought were a desirable potential medical tourism inflow were visitors who routinely stayed for long periods at a time, up to two to three months per year, many of whom own or rent long-stay vacation properties. This group of tourists were thought to be “*unique*” to Barbados, giving it an advantage over other countries. Short-stay repeat visitors were viewed as an important existing and future medical tourism market as well, including “*people who’ve been in Barbados and have come to Barbados for the last twenty years and keep coming*.”

As per participants’ understandings, Barbados’ existing base of recreational tourists were being reframed as a readily accessible inflow of future medical tourists. The appeal of these recreational tourists as potential medical tourists was found in the fact that they were already visiting Barbados and therefore struck stakeholders as a realistic and achievable patient base. Whereas other types of medical tourists must be encouraged to visit Barbados in the first place, this group of potential medical tourists were already largely familiar with the island. As one participant put it, they were an *“easy win for medical tourism*.”

### Targeting established inflows as future medical tourists: regional LAC patients

Stakeholders from both countries viewed privately paying patients from around the LAC region as an important part of existing revenue in the private health care sector and also an important part of an expanded medical tourism sector. For example, a Barbadian participant stated that “*there’s three and half million people around the Caribbean who want that kind of care …across the Caribbean [the] patients who travel here is immense*.” Others described the LAC region as “*a huge market*” or a “*key market*”. Many participants from both countries were quick to point out that Barbados and Guatemala alike would compete for these patients against other countries in the LAC region and so would need to be strategic in expanding upon this existing patient inflow. Further to this, the stakeholders we spoke with cited the comparatively high prices for private health care in Barbados as a barrier to further serving this highly desirable patient group.

Two reasons were most commonly provided as to why people from elsewhere in the LAC region would travel to either Barbados or Guatemala, and these same reasons were shared by participants from both countries. The first is to access better quality of care than is available at home. A participant involved in planning a private medical facility in Barbados spoke of the country’s potential to serve as a “*high quality option for regional health care*” because it “*historically has had a reputation regionally for better health care*” than many of its neighbouring countries. Some interviewees stated that Guatemala had superior quality of medical services in all areas compared with neighboring areas such as El Salvador and Southern Mexico, while others discussed its competitive advantage in certain sub-disciplines. The second reason given for intra-regional medical travel by LAC regional patients was to access care not available at home. Guatemala, for example, was cited as having “*more and better specialists than in their own countries*” and people from the region “*have faith in the Guatemalan physicians*.” A Barbadian stakeholder reported that in some countries in the Eastern Caribbean, residents have “*no choice* [for care] *and therefore they came here*.”

### Targeting New inflows as future medical tourists: un/under-insured americans

Participants from both countries spoke of the perceived potential of attracting un- or under-insured American patients to their countries as a way to expand their medical tourism sectors. The largely private nature of the US health system was seen as creating a pool of potential customers as “*there are 50 million uninsured Americans. If you can’t pay for insurance, you cannot pay for a medical service in the States*.” Many participants discussed projections for potential US patients that were extremely optimistic and based on the country’s relatively large and aging population. For example, a Guatemalan participant referred to a report created by the Deloitte consultancy [40] that is widely cited throughout the global medical tourism industry, explaining “*that’s been fundamental for all of us in Guatemala*” in understanding the medical tourism market in the US. This report was interpreted as claiming that at least 9 million people would soon be traveling from the US for medical care each year. Even under this “*pessimistic*” calculation, one participant exclaimed that “*that’s enough patients for everyone!*” Such comments were echoed by Barbadian participants, one of whom said that the planned AWC facility was “*the biggest thing for Barbados*” in terms of attracting this inflow. Changes to the US health system through the Affordable Care Act (ACA) were viewed as a factor participants thought would push patients out of the US and into their medical tourism sectors, with some suggesting that Americans remaining uninsured may opt for care abroad while those who are under insured may find care abroad more affordable than domestic options depending on the costs of their co-payments.

Within both countries, there was a firm belief that US patients can be attracted abroad on the basis of price compared to that of care available domestically. One Guatemalan participant suggested cost savings in Guatemala up to 75 % off US prices for particular procedures. Similarly, patients from the US would be drawn by the lower costs of care in Barbados, where the “*cheaper version*” of the same medical procedures could be accessed for “*a quarter*” of the price. Retirees from the US were seen as an additional growth market in Barbados as medical care there was understood to be less expensive than that in the US. Participants also discussed the potential of coupling low prices with insurance reimbursements for American customers, including “*some kind of a formal working agreement*” with US insurance companies. In this way, American patients would be able to travel to Guatemala and Barbados for care, with insurance companies subsidizing or paying fully. Offering such arrangements was viewed to be a way to attract greater inflows of (under) insured American patients. Some Guatemalan participants were wary of the viability of establishing this particular inflow, though, suggesting that the country’s high rates of violence would lessen its attractiveness to American patients being given multiple international care options by their insurers: “*if I was an American, and I was offered to come to Guatemala, I wouldn’t really like to come here. Maybe the price would be tempting… but violence is so terrible here*.”

### Targeting New inflows as future medical tourists: North Americans seeking more accessible care

Regardless of patients’ insurance status at home, it was recognized by participants in both Barbados and Guatemala that their medical tourism inflows would grow by offering care that is more accessible, either cost wise or timeliness wise, to Canadian and American patients alike. For attracting inflows of Canadian patients, it was recognized that the price of services mattered but also the speed at which they could be accessed. Due to the public nature of the Canadian health system, these patients were commonly perceived as facing wait times for their care that could be overcome by traveling to Guatemala or Barbados. As one Guatemalan participant noted, in Canada “*the problem is not the lack of services but the fact that services are over saturated so in order to fix someone’s knee problem in a surgery, it would take over two years to get it scheduled*.” More generally, participants from both countries widely acknowledged that there are people in both Canada and the US who are looking abroad to access care to avoid long wait times or high costs at home and that attracting such patients into their medical tourism markets was highly desirable.

The view that Barbados and Guatemala could compete for North American patients seeking more accessible care on the basis of cost specifically, rather than those seeking care that is timelier, was thrown into doubt by some participants. Several Barbadians identified Barbados as having a relatively high cost of care, even when compared with the US. While medical care in Guatemala was generally thought to be considerably less expensive than that in the US and Canada, it was noted that there are countries elsewhere in the LAC region with even lower costs, thus placing Guatemala at a competitive disadvantage. A participant argued that “*we shouldn’t waste our time in going to the US to promote and sell our packages – even if American Airlines gives us free tickets – because we are more expensive than the rest of the region and no one will want to come.*” The distance of both Guatemala and Barbados from Canada and the US was seen as problematic to capturing this inflow as well. One participant expressed doubt about Barbados’ potential in recruiting such patients, saying “*I don’t see anyone flying from States or Canada to come to Barbados for treatment*.” The lack of English language competency in Guatemala was seen as “*the biggest barrier*” to accessing this inflow for the country.

### Targeting New inflows as future medical tourists: North Americans seeking procedures not available at home

Participants in both Guatemala and Barbados widely thought that patients from the US and Canada in particular would be motivated to travel abroad for care not available domestically, such as experimental procedures and procedures still undergoing approval by the US Food & Drug Administration (FDA) or other regulators. As a result, it was felt that the growing medical tourism sectors in these countries could capitalize on such demand. As these countries are not limited by US or Canadian regulators in what treatments they can offer, they can use their presence outside of these legal systems as a competitive advantage, providing North American patients with a reason to travel abroad for care.

Stakeholders thought North American patients might be motivated to travel abroad to access these experimental treatments out of a sense that conventional treatments are not likely to be as successful, because all conventional treatments have been exhausted, or because of a sense that one’s health is deteriorating while the patient waits to access new treatments domestically. One Guatemalan participant noted that these factors are highly motivating for patients and that “*there is a sense of urgency in the search of this* [stem cell] *treatment*” in particular. The availability of procedures not approved in Canada and the US was seen as a draw for Barbados as well, as in the past Barbados had offered “*certain orthopaedic procedures that were not licensed to be carried out in America*.” Offering unapproved interventions was seen as a potentially lucrative market for both countries. As a Guatemalan stakeholder described it, “*people are more committed and…due to its cost they leave a considerable profit*.” While North American regulators will grant approval for some of these treatments offered abroad, the stakeholders we spoke with indicated that they could adapt to these changes, offering new treatments as they are developed.

## Discussion

Participants in Barbados and Guatemala identified numerous comparative advantages and regional patient markets that were thought to provide for success in the medical tourism market. Each country currently receives a small number of LAC regional patients as customers for their existing private medical sectors. These regional markets are different in each case, with Guatemala drawing on Spanish-speaking countries in Central America, Southern Mexico, and the Western Caribbean and Barbados drawing from English-speaking countries in the Eastern Caribbean. While each country must compete with other proximal nations for these patients, the diversity and size of the LAC region allows for sub-regional competition and specialization in how countries market themselves to medical tourists. Moreover, the large size of the Guatemalan diaspora provides another market that Guatemala has privileged access to over other countries in the region. This large diaspora was, according to participants, an important customer base that allows Guatemala to overcome considerable reputational problems, especially around violence in the country. While Barbados has a smaller and, according to our participants, less important diaspora to draw on for customers, its positive international reputation as a safe tourism destination, existing tourism inflows, and English-speaking population give it preferred access to specific markets within the region and in North America as well. Importantly, stakeholders did not see all of these markets in terms of ‘medical tourism’, particularly in the case of diaspora and regional patients. The label of ‘medical tourist’ was most consistently applied to non-readily accessible patients whereas more readily accessible patients were more generally seen simply as internationally-based patients seeking private care.

### Medical tourism dreams and reality

There is a danger that these countries’ interests in expanding their medical tourism sectors and gaining greater exposure in the North American market are unlikely to be as successful as their participation in LAC regional and diaspora markets. Of particular concern are the expectations and factual claims raised by some of the stakeholders we interviewed. For example, one Guatemalan stakeholder based their expectations of being able to enhance inflows of diaspora patients on there being 1.8 million Guatemalans in California alone and “*a lot of Guatemalans*” in Canada. In reality, in 2010 there were 332,737 persons of Guatemalan origin in California [41], 1.l million in the US overall [42], and 16,150 Guatemalan immigrants living in Canada in 2006 [43]. While these numbers point to a significant Guatemalan population in the US at least, they are far below this stakeholder’s estimates. Similarly the 2008 Deloitte consultancy report [40] referenced as being “*fundamental to all of us in Guatemala*” in reporting that “*9 million*” Americans would be going abroad for medical tourism was itself heavily revised downward in 2009 to 1.6 million medical tourists estimated for 2012 [44]. While stakeholders felt that the ACA and US employer insurance would create new flows of patients from the US, the updated Deloitte report indicates that the effects of the ACA on employer benefits and health insurance are “uncertain” and no updated report has been developed since the passage of the ACA [44]. Beliefs about wait times for care driving Canadian patient flows were similarly unrealistic. While one stakeholder expressed that Canadians must wait “*over 2 years*” to receive knee surgery, in reality half of Canadians referred for such procedures receive this care in 182 days and 90% within 258 days [45]. Importantly, these optimistic views were not universally held, with some stakeholders in both Guatemala and Barbados doubting that they would be cost competitive with established LAC regional medical tourism exporters, including Mexico (which is closer to the US) and Costa Rica (which is considered safer than Guatemala).

The extremely optimistic estimates of international patient flows by many of the stakeholders we spoke with may be the result of optimism following their decision to diversify their tourism markets or a failure to fully inform themselves about the reality of medical tourism. External actors are also certainly to blame for this potentially unrealistic view of the medical tourism market as well [18]. The aforementioned Deloitte consultancy report, which was consistently misread in favor of medical tourism development by the stakeholders we spoke with, has itself been criticized for presenting an overly optimistic view of future sector growth [46]. Industry members have made a habit of visiting LAC countries to promote investment in the medical tourism sector as well. For example, the Medical Tourism Association, an industry group that promotes and facilitates sector development, has held meetings promoting medical tourism development in Antigua [47], Barbados [48], Guyana [49], Puerto Rico [50], St. Lucia [51], and other countries in the region in recent years. Many of these and other LAC countries also regularly attend Medical Tourism Association sponsored events such as the World Medical Tourism and Global Healthcare Congress held annually in the US [52]. Non-industry groups have actively promoted the medical tourism sector in the LAC region as well. For example, the Caribbean Export Development Agency (CEDA) and Canadian Trade Facilitation Office hosted a regional meeting in Barbados in 2008 aimed at increasing health sector exports to Canada [24, 53]. CEDA continues to be active in promoting the health and wellness sector in the Caribbean by developing strategy documents for this related sector [54]. Importantly, some stakeholders noted that their countries were not clearly cost competitive with other, established medical tourism destinations in the region and doubted that patients would flock to these countries in the numbers estimated by other stakeholders. It is not clear how well these dissenting voices are being heard by local policy makers, however, given that each country continues to proceed with plans for promoting their domestic medical tourism sectors.

### The costs of pursuing the medical tourism dream

Given that some stakeholders with whom we spoke systematically overestimated the likely medical tourism flows from North America and misinterpreted or relied on outdated versions of industry reports, there is real cause for concern that the expectations driving development of the medical tourism industry in these countries will not match the reality of future patient flows, and especially the new inflows they seek to develop. Developing a medical tourism sector creates opportunity costs for these countries, taking time and resources away from government ministries and medical professionals who are drawn into planning, regulation, and facilitation of this sector [16, 55]. These groups have finite resources, and time spent promoting and planning for the development of the medical tourism sector necessarily means time not put to addressing pressing local health needs. Development deals for medical tourism facilities also often include land lease deals, redirecting resources into this sector that might have been put to other purposes. For example, Barbados agreed to lease a former public hospital site to the AWC developers for up to 50 years, meaning that this site could not be re-developed for local or non-medical use [56].

Development of the medical tourism sector creates costs and vulnerabilities in absolute terms as well. Though not explored in this analysis, many of the stakeholders we spoke with discussed a push into seeking international accreditation in order to attract international patients. Such accreditation is expensive and time consuming [21, 57], and if international patients do not come in numbers justifying these expenses, then both public and private medical providers will lose out on recouping such investment. Given high levels of competition throughout the LAC region for investment in medical tourism, developers may seek concessions from countries that include changes to malpractice laws, health worker licensure, and duties on imported medical supplies and equipment that may disadvantage local medical providers [3, 16, 21]. In the case of Barbados, AWC has responded to the global economic downturn by demanding loan guarantees from Barbados while simultaneously exploring alternative developments in other Caribbean countries [58]. Should these and other developments founder due to overly optimistic estimates of inflows of international patients (particularly Americans), destination countries such as Guatemala and Barbados will be left with these potentially enormous costs and little to show for them.

### Responsibility for promoting the medical tourism dream

Given the potential costs of pursuing medical tourism development, there is an important question of whether industry groups should be actively promoting the medical tourism sector in the LAC region. It is not surprising that these groups are actively encouraging development of the medical tourism sector across the region as they stand to gain financially from doing so, either by offering training, certification, and marketing services to governments [59] or as a way of promoting their consulting businesses. However, if their success relies on intentionally or even negligently overestimating the likely North American patient flows to LAC destinations, then these efforts are morally problematic. Moreover, if industry groups such as the Medical Tourism Association make similar pitches of the promise of medical tourism to countries throughout the region, then they should be held morally accountable if, in competing for this same group of customers, many of these countries find themselves losers in the regional race for success in the medical tourism market.

Also troubling are efforts by non-industry groups such as CEDA and the Canadian Trade Facilitation Office to encourage medical tourism development in the LAC region. Whereas the mission of groups like these is to promote trade in the region, if patient flows do not match the high expectations of the stakeholders we spoke with, there is a danger that these groups will merely encourage unsustainable levels of investment and oversaturation of the private medical market. These actions, rather than promoting new avenues for economic development, could instead hinder the provision of health services within these countries if these countries divert attention from providing domestic health services to promoting private care for international patients and direct resources into promoting this industry and attracting foreign direct investment.

### Grounding the medical tourism dream in reality

Despite the dangers posed to Barbados and Guatemala of pursuing a potentially unrealistic flow of patients from the US, Canada, and other markets, these countries do have the potential to expand their medical tourism markets. More clearly promising medical tourism markets exist for Barbados and Guatemala in areas where these countries have a competitive advantage over other LAC competitors. For Barbados, existing flows of recreational tourists offer a source of potential patients that could be expanded on. In Guatemala, the large diaspora population abroad is also worth attempting to develop as a patient base. Both countries also have bases of regional patients and can provide treatments unapproved in North America, though regional competition for patients in both of these areas is great and this market introduces new risks including reputational harms if these treatments are not well regulated and are taken advantage of by fraudulent service providers [60]. These expansion efforts, if pursued, should be grounded in careful, independent research of market potential rather than the potentially overly optimistic boosterism of those with an interest in promoting this sector.

Other LAC countries face similar pressures to invest in the medical tourism market and challenges in successfully doing so [1, 3]. As this study has shown, the perceived opportunities for successful medical tourism expansion differ between LAC countries. Similarly, pressures on medical tourism expansion and the expectations for where future medical tourism customers will flow from differ between different countries based on their geography, culture, and history. Therefore, additional research is needed into the past development of and future plans for the medical tourism sectors in all LAC countries. This research will be crucial to helping to reign in the medical tourism dream, promoted by industry and non-industry groups alike, in these countries based on likely future patient flows. Moreover, a better understanding of the context of specific LAC countries will make regional cooperation possible, ensuring that these countries are not simply undercutting one another in pursuit of the same customer base. This research is the only way to ensure that pursuit of the medical tourism dream in LAC countries does not result in significant negative consequences should this dream not be realized.

## Conclusion

Barbados and Guatemala each have relied on patients from neighbouring LAC countries to support their nascent medical tourism sectors. Each has also found a competitive advantage in this sector as well, with Barbados taking advantage of its positive reputation as a tourist destination and English speaking population and Guatemala drawing on its large diaspora population. These countries, along with many others in the region, are also seeking to expand their medical tourism sectors by attracting new patient inflows from the US and Canadian markets. Success in these markets is uncertain as the expectations of patient flows expressed by the 100 stakeholders we interviewed for this analysis were often not backed by the reality of the US and Canadian medical tourist patient markets.

There is a danger, then, that the medical tourism ‘gold rush’ in the LAC region will create substantial costs to those countries that invest in the development of this sector if expected patient flows do not materialize. Almost certainly some LAC countries will benefit from development of the medical tourism sector. Rather than competing with one another for the same group of patients and by using the same marketing plan, the differences between and comparative advantages of these countries should be emphasized [13]. While there is no way to ensure widespread benefits across the LAC region from developing the medical tourism sector, we believe these countries would benefit from cooperation with one another and strategic planning around specialization and targeting of sub-populations of patients. While cooperation of this kind is extremely difficult to achieve, without it the danger is that these countries will each continue to dream about attracting the same new international patient inflows, not realizing that this dream may not be grounded in reality.

## Abbreviations

ACA: Affordable Care Act
AGEXPORT: Guatemalan Association of Exporters
AMC: American World Clinics
CEDA: Caribbean Export Development Agency
FDA: Federal Drug Administration
LAC: Latin American and Caribbean
UK: United Kingdom
US: United States
